# Unspecified asthma, childhood-onset, and adult-onset asthma have different causal genes: a Mendelian randomization analysis

**DOI:** 10.3389/fimmu.2024.1412032

**Published:** 2024-11-19

**Authors:** Roan E. Zaied, Sreemol Gokuladhas, Caroline Walker, Justin M. O’Sullivan

**Affiliations:** ^1^ The Liggins Institute, The University of Auckland, Auckland, New Zealand; ^2^ Faculty of Medical and Health Sciences, The University of Auckland, Auckland, New Zealand; ^3^ The Maurice Wilkins Centre, The University of Auckland, Auckland, New Zealand; ^4^ Australian Parkinsons Mission, Garvan Institute of Medical Research, Sydney, NSW, Australia; ^5^ MRC Lifecourse Epidemiology Unit, University of Southampton, Southampton, United Kingdom; ^6^ Singapore Institute for Clinical Sciences, Agency for Science Technology and Research, Singapore, Singapore

**Keywords:** Mendelian randomization, childhood-onset asthma, adult-onset asthma, gene regulatory networks, SNP function, genetic risk, expression quantitative trait loci

## Abstract

**Introduction:**

Asthma is a heterogeneous condition that is characterized by reversible airway obstruction. Childhood-onset asthma (COA) and adult-onset asthma (AOA) are two prominent asthma subtypes, each with unique etiological factors and prognosis, which suggests the existence of both shared and distinct risk factors.

**Methods:**

Here, we employed a two-sample Mendelian randomization analysis to elucidate the causal association between genes within lung and whole-blood-specific gene regulatory networks (GRNs) and the development of unspecified asthma, COA, and AOA using the Wald ratio method. Lung and whole blood-specific GRNs, encompassing spatial eQTLs (instrumental variables) and their target genes (exposures), were utilized as exposure data. Genome-wide association studies for unspecified asthma, COA, and AOA were used as outcome data in this investigation.

**Results:**

We identified 101 genes that were causally linked to unspecified asthma, 39 genes causally associated with COA, and ten genes causally associated with AOA. Among the identified genes, 29 were shared across some, or all of the asthma subtypes. Of the identified causal genes, *ORMDL3* had the strongest causal association with both unspecified asthma (OR: 1.49; 95% CI:1.42-1.57; p=7.30x10^-51^) and COA (OR: 3.37; 95% CI: 3.02-3.76; p=1.95x10^-102^), whereas *PEBP1P3* had the strongest causal association with AOA (OR: 1.28; 95% CI: 1.16-1.41; p=0.007).

**Discussion:**

This study identified shared and unique genetic factors causally associated with different asthma subtypes. In so doing, our study emphasizes the need to move beyond perceiving asthma as a singular condition to enable the development of therapeutic interventions that target sub-type specific causal genes.

## 1 Introduction

Asthma is a chronic disease of the airways that impacts an estimated 300 million people worldwide (1, 2). Recent studies suggest that asthma is not a single disease but rather a multifaceted spectrum of disorders characterized by shared features, including reversible airway obstruction, wheezing, and cough (2–6). Despite this, asthma studies, including Genome-Wide Association Studies (GWASs), predominantly employ a broad definition often referred to as unspecified asthma (7–9). The use of this definition is attributed to reasons including complexities in identifying and categorizing various asthma subtypes and challenges in recruiting sufficient participants representing said subtypes to achieve statistical power (10, 11).

Childhood-onset asthma (COA) and adult-onset asthma (AOA) are prominent asthma subtypes. Apart from their differences in age of onset, COA and AOA diverge in their etiology, clinical severity, comorbidities, response to treatment, and prognosis (12–14). COA is mostly associated with perinatal factors, respiratory tract infections, and atopy, whereas AOA often needs higher doses of corticosteroid treatment, tends to be non-allergic, and is often linked to environmental and lifestyle elements such as smoking and obesity (2, 15). Notably, a familial history of asthma or allergic conditions (*e.g.*, rhinitis and eczema) is a stronger predictor of COA than AOA (2, 16). Collectively, these observations indicate the presence of specific risk factors that are associated with age of asthma onset (*i.e.*, childhood or adulthood).

GWASs have identified shared and distinct genetic risk loci associated with unspecified asthma, COA, and AOA (8, 14, 17–19). However, the functional interpretation of these identified SNPs remains challenging. This is due to SNPs’ subtle effect sizes and the fact that more than 90% of GWAS SNPs fall in non-coding regions of the DNA (20). Moreover, insights from genetic studies, including expression quantitative trait loci analysis (eQTL), have shown that many disease-associated SNPs participate in long-range interactions, complicating standard gene-target identification methods such as the “nearest gene approach” (21–23).

The aims of this study are to investigate the distinct and shared genetic risk factors contributing to the development of unspecified asthma, COA, and AOA. Specifically, we seek to identify the genes that are causally associated with each asthma subtype and their relevant pathways, which will help prioritize them for future research (8, 24). This prioritization will present new opportunities for asthma drug development and repurposing. Furthermore, exploring causally associated genes and pathways that are shared, or distinct among asthma subtypes could provide insights into the importance of considering asthma subtypes in refining therapeutic strategies.

To this end, we employed Mendelian randomization (MR), a statistical analysis that relies on the random assortment of genetic variants at conception to simulate randomized controlled trials. MR enables the inference of causal risk associations between risk factors (exposures) and disease outcomes under specific assumptions (25, 26). By utilizing genetic variants as instrumental variables (IVs), MR can mitigate the impact of confounding factors and reverse causation (27). In this study, we applied MR to identify whether genes regulated by tissue-specific spatial eQTLs (exposures) are causally associated with the development of unspecified asthma, COA, and AOA (outcomes). Accordingly, our analysis identified putative spatial eQTL-gene pairs that causally influence each asthma subtype, suggesting potential for more targeted approaches in asthma care.

## 2 Methods

### 2.1 Generation of the lung and blood gene regulatory networks

Gene regulatory networks (GRNs) that are lung (L-GRN) and whole-blood (B-GRN) specific were generated as outlined in (28). Briefly, all SNPs called in the Gene Tissue Expression (GTEx) project (21) (n = ~40 million, MAF ≥ 0.05, dbGaP accession: phs000424.v8.p2; project number: #22937) were run through the CoDeS3D pipeline (https://github.com/Genome3d/codes3d-v2) (29). Restriction enzymes (HindIII and MboI) were used to digitally digest the hg19 genome into SNP-harboring DNA fragments. CoDeS3D, in turn, queried Hi-C chromatin interaction libraries for primary lung (30) and blood samples (31, 32) to identify SNP-harbouring DNA fragments that physically interact with gene-harbouring DNA fragments. A chromosome-based multiple testing correction (Benjamini-Hochberg) was then performed to identify significant (adjusted p-value ≤0.05) tissue-specific spatial eQTLs (eQTLs that physically interact with their target genes through chromatin looping (33)) and their tissue-matched target genes. The resulting L-GRN and B-GRN thus consist of spatial eQTL-gene pairs from their respective tissue. Both GRNs are available online.

### 2.2 Mendelian randomization

The *TwoSampleMR* package (v0.5.6) (31) was used to identify if genes (exposures) within the L-GRN and B-GRN were causally associated with the development of three asthma subtypes (outcomes) namely 1) asthma defined using a broad definition (*i.e*., self-reported asthma or ever having had a doctor’s diagnosis of asthma), hereafter referred to as unspecified asthma, 2) COA, and 3) AOA, using spatial eQTLs as IVs. A genetic variant must satisfy three pivotal assumptions to be included in the MR analysis. Genetic variants must (i) exhibit strong associations with the exposure, (ii) genetic variants must be associated with the outcome through the tested exposure only, and (iii) have no associations with any potential confounders that affect the outcome. Genetic variants meeting those assumptions are termed IVs. Spatial eQTL-(p-value < 1x10^-5^) gene pairs from each GRN were used as the exposure dataset, where only independent SNPs were retained using linkage disequilibrium (LD) clumping. Clumping was performed using the 1000 genome project (Phase III) European reference panel (clumping window = 10,000, r^2^ < 0.001).

GWAS summary statistics from Valette et al. (including 56,167 cases [mean age of 56.5, 42.5% males] and 352,255 controls [mean age of 57.0, 46.4% males]) were used as outcome data for unspecified asthma (8). These include patients with a diagnosis from hospital or primary care records and those with self-reported asthma (8). GWAS summary statistics from Zhu et al. were used as outcome data for both COA (age of onset ≤12 years) and AOA (age of onset ≥26 years), including a total of 9,676 COA cases, 22,296 AOA cases, and 347,481 shared controls (14). The GWASs included in this analysis were all conducted using the UK Biobank cohort, and the analysis was restricted to individuals of European ancestry (8, 14).

IVs that are not present in the outcome GWAS were replaced with their proxy SNPs (r^2^ ≥ 0.6). IVs that are absent and have no proxy SNPs in the outcome GWAS were excluded from the analysis. To ensure that a spatial eQTL’s effect on an exposure (*i.e.*, genes) and outcome (*i.e.*, development of asthma subtype) correspond to the same allele, the exposure and outcome data were harmonized using the *harmonise_data()* function. MR analysis was then performed to estimate the causal effect of exposures having a single IV using the Wald ratio method (Bonferroni-corrected, p < 0.05/number of exposures).

For exposures having multiple IVs, only those passing a sensitivity analysis were retained. The presence of heterogeneity (dispersion of SNP effects) suggests a violation of IV assumptions, with horizontal pleiotropy likely being the primary contributor (27). Accordingly, we have conducted a heterogeneity test using Cochran’s Q statistic and excluded exposures whose IVs have significant heterogeneity (Q_pval < 0.05) from further analysis. Moreover, we also used the MR-Egger intercept term (*mr_pleiotropy_test()* function) to assess for horizontal pleiotropy, which is said to be present when the MR-intercept term significantly deviates from the null (p < 0.05) (34). The inverse variance weighted (IVW) method was then used to estimate the causal effects of exposures having multiple IVs (Bonferroni-corrected, p < 0.05/number of exposures). All results correspond to an odds ratio (OR) of asthma subtype per 1-SD change in gene expression in the lung or whole blood.

Custom tracks on the UCSC Genome Browser (https://genome.ucsc.edu/cgi-bin/hgCustom, GRCh38) were used to visualize spatial eQTLs and their target genes.

### 2.3 Biotype annotation of causal asthma genes

Biotype annotation (*e.g.*, protein-coding, pseudogenes, lincRNA, etc.) of the genes causally associated with unspecified asthma in lung and blood was achieved using the GENCODE database (http://ftp.ebi.ac.uk/pub/databases/gencode/Gencode_human/release_26/gencode.v26.annotation.gtf.gz, accessed on 05/06/2021).

### 2.4 Pathway enrichment analysis

Genes causally associated with unspecified asthma, COA, or AOA, were tested for gene ontology (GO) enrichment using the *gost()* module from the *gprofiler2* R package (35). Genes were assessed for enrichment in biological processes, molecular functions, cellular components, and the Kyoto Encyclopedia of Genes and Genomes (KEGG) pathways (36). Ontology terms and pathways with a Benjamini-Hochberg (37) corrected p-value ≤ 0.05 were considered statistically significant.

### 2.5 Identification of immune cell types and tissues having shared regulatory patterns of causal asthma genes

Spatial eQTL-gene pairs causally associated with unspecified asthma identified using MR were mapped onto 15 immune cell types (*i.e*., innate immune cell types [natural killer cells [NK], classical monocytes, and non-classical monocytes [M2 cells]], naïve adaptive immune cells [naïve B, naïve CD4+ T cells, activated naïve CD4+ T cells [CD4_stim], naïve CD8+ T cells, activated naïve CD8+ T cells [CD8_stim], and naïve TREG cells], and CD4+ T memory subtypes [Memory TREG, TH1, TH 1/17 [TH star], TH17, TH2, and follicular helper T cells [TFH]]) from the Database of Immune Cell Expression, Expression of quantitative trait loci and Epigenomics (DICE, https://dice-database.org/downloads/, accessed 09/02/2021) (21). The *pheatmap* package (version 1.0.12) was used to perform unsupervised hierarchical clustering of eQTL effect sizes (beta) obtained from DICE across the 15 DICE immune cell types.

## 3 Results

### 3.1 eQTL analysis identifies spatially constrained eQTLs in the lung and blood

**Figure 1:**
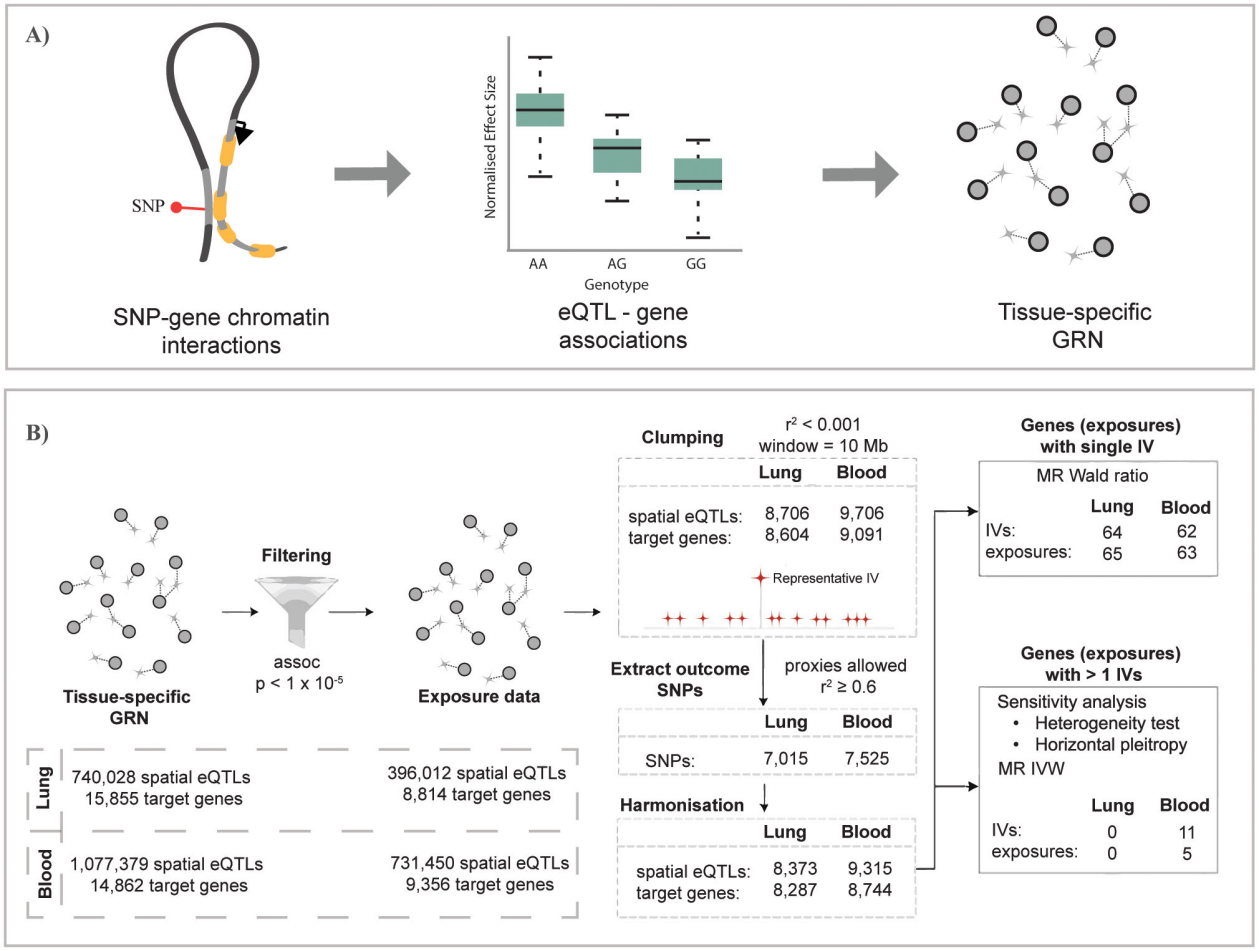
Overview of the study pipeline. **(A)** Construction of gene regulatory networks for GTEx lung and whole-blood tissues using the CoDeS3D pipeline (29). **(B)** Summary of the Mendelian randomization analysis used to identify genes causally associated with unspecified asthma in lung and blood (the same analysis was conducted for COA and AOA). **(A)** is adapted from Zaied et al. (28).

We combined chromatin interaction data (Hi-C) and eQTL data from GTEx to build tissue-specific spatially constrained GRNs in which putative functions are assigned to SNPs by associating them with their target genes (**Figure 1A**) (29). The resulting L-GRN contains 740,028 spatial eQTLs regulating the expression of 15,855 genes in the lung, constituting 873,133 spatial eQTL-gene interactions. The B-GRN has 1,077,379 spatial eQTLs regulating the expression of 14,871 genes in whole blood, making a total of 1,713,885 spatial eQTL-gene interactions. The L-GRN and B-GRN have been described extensively in Zaied et al. (28).

### 3.2 Mendelian randomization identifies genes causally associated with unspecified asthma in the lung and blood

**Figure 2:**
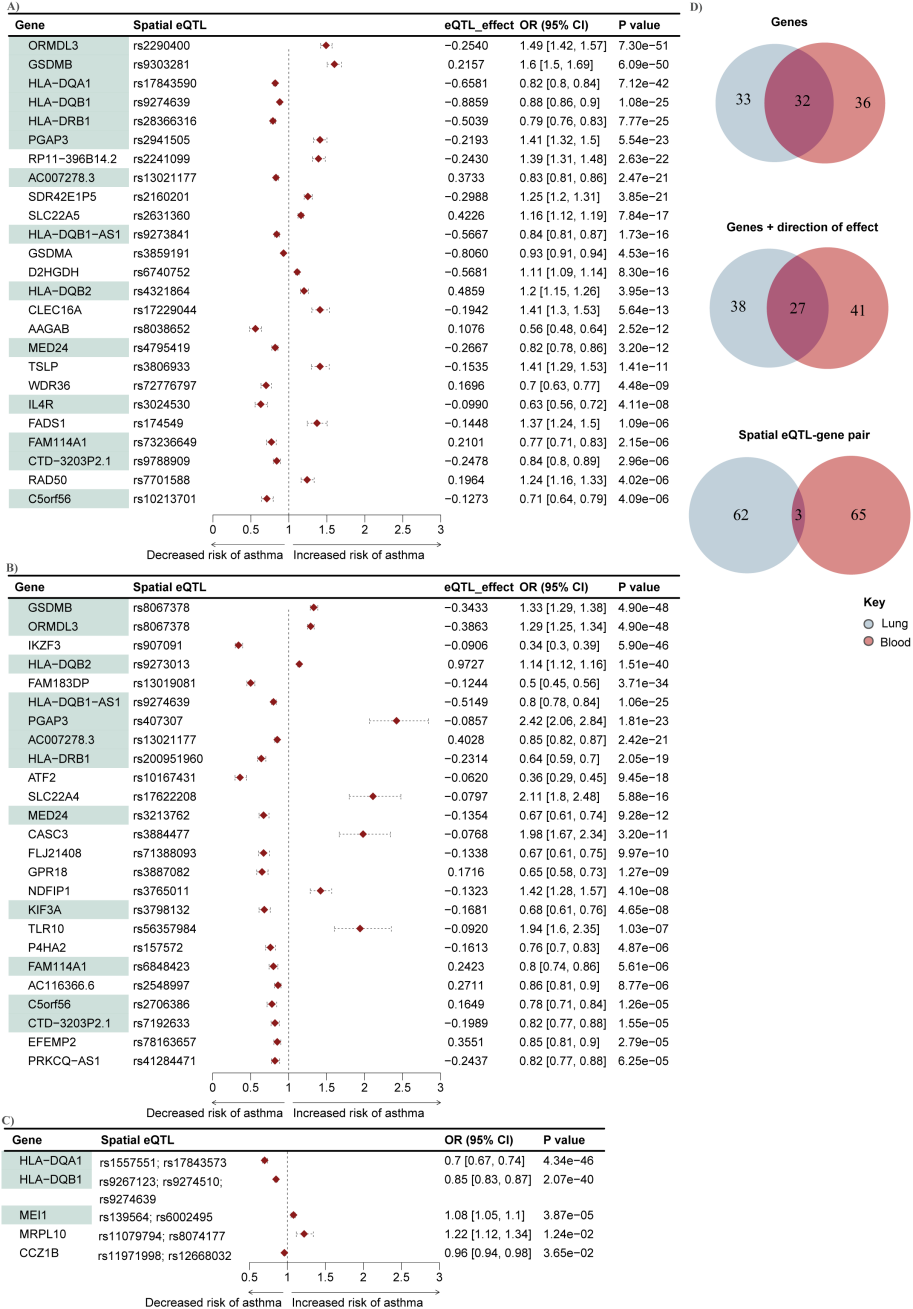
Mendelian randomization identifies 69 lung or blood-specific genes and 32 shared genes that are causally associated with unspecified asthma. 65 and 63 genes (exposures) causally associated with unspecified asthma were identified in the **(A)** L-GRN and **(B)** B-GRN using the Wald ratio method, respectively. Only the top 25 (ranked by p-value) genes and their spatial eQTLs (IVs) are shown here. A complete list of the genes we identified as being causally associated with asthma is presented in **Supplementary Tables 1 and 2**. Genes (n = 32) shared across both GRNs are shaded in green. Horizontal dotted lines illustrate the 95% CI for each odds ratio. **(C)** Five genes with multiple IVs (inverse variance weighted method) were identified as causally associated with unspecified asthma in the blood. **(D)** 32 genes were shared between the lung and blood (top). Of those shared genes, 27 had the same direction of effect (odds ratio) on unspecified asthma (middle), and only three were regulated by the same spatial eQTL in both tissues (bottom).

We conducted a two-sample MR analysis to test whether L-GRN and B-GRN genes were causally associated with the development of unspecified asthma using spatial eQTLs as IVs (**Figure 1B**). Among exposures with single IVs, the Wald ratio method identified 65 L-GRN genes regulated by 64 spatial eQTLs whose altered expression in the lung is causally associated with unspecified asthma (**Figure 2A**, **Supplementary Table 1**). No exposures with multiple IVs were identified to be causally linked to unspecified asthma in the L-GRN. Of the 65 genes causally associated with unspecified asthma in the lung, 43 were protein-coding, and 22 were non-coding biotypes, *e.g.*, antisense [n=10] and pseudogenes [n=6], etc., **Supplementary Figure S1**). These genes were enriched for 17 pathways in KEGG, including asthma, inflammatory bowel disease, and Th1 and Th2 cell differentiation (**Supplementary Figure S2A**).

In the B-GRN, the altered expression of 63 genes regulated by 62 spatial eQTLs was identified as being causally associated with unspecified asthma using the Wald ratio method (**Figure 2B**). Moreover, the altered gene expression of five genes regulated by 11 spatial eQTLs was identified as causally associated with unspecified asthma using the IVW method (**Figure 2C**, **Supplementary Table 2**). 53 of the genes identified as causally associated with unspecified asthma in blood were protein-coding, and 15 were non-protein-coding, *e.g.*, antisense [n=8] and processed transcripts [n=3] (**Supplementary Figure S1**). The identified causal genes in blood were enriched for 12 KEGG pathways, including Th1 and Th2 cell differentiation, asthma, and Th17 cell differentiation (**Supplementary Figure S2B**). Among the protein-coding genes causally associated with unspecified asthma in lung and blood, seven are within the 17q12-21 locus (*i.e*., *PGAP3*, *IKZF3*, *GSDMB*, *ORMDL3*, *GSDMA*, *MED24*, and *CASC3*; **Supplementary Figure S3**).

Amongst the putatively causal genes we identified, 69 were lung or blood-specific, and 32 were shared between the L-GRN and B-GRN. Five of the 32 shared genes had opposite directions of effects in either tissue. Specifically, three genes were associated with increased asthma risk in the lung and a decreased risk for asthma in the blood (*i.e.*, *RERE*, *UNC13D*, and *OIP5-AS1*), and two genes (*i.e., RP5-1115A15.1* and *DEF6*) had the opposite effect. The remaining 27 shared genes had the same direction of effect on asthma risk in both tissues, where only three of those genes were regulated by the same spatial eQTL across tissues (**Figure 2D**).

Spatially constrained eQTLs are a critical component of the process of identifying putative asthma causal genes in this study. Of the genes that were identified as being causal in lung and blood, all were regulated in cis (spatial eQTLs located within +/-1 Mb of their target genes on the same chromosome) except for three spatial eQTL-gene pairs (*i.e.*, rs10167431-*ATF2*, rs9267123-*HLA-DQB1*, and *rs61044849-RABL3*) which were regulated by trans-intrachromosomal eQTLs (spatial eQTLs located >1 Mb away on the same chromosome; **Supplementary Table 2**).

### 3.3 The shared regulatory patterns of causal asthma genes across different immune cell types suggests similar effects on the development of asthma

**Figure 3:**
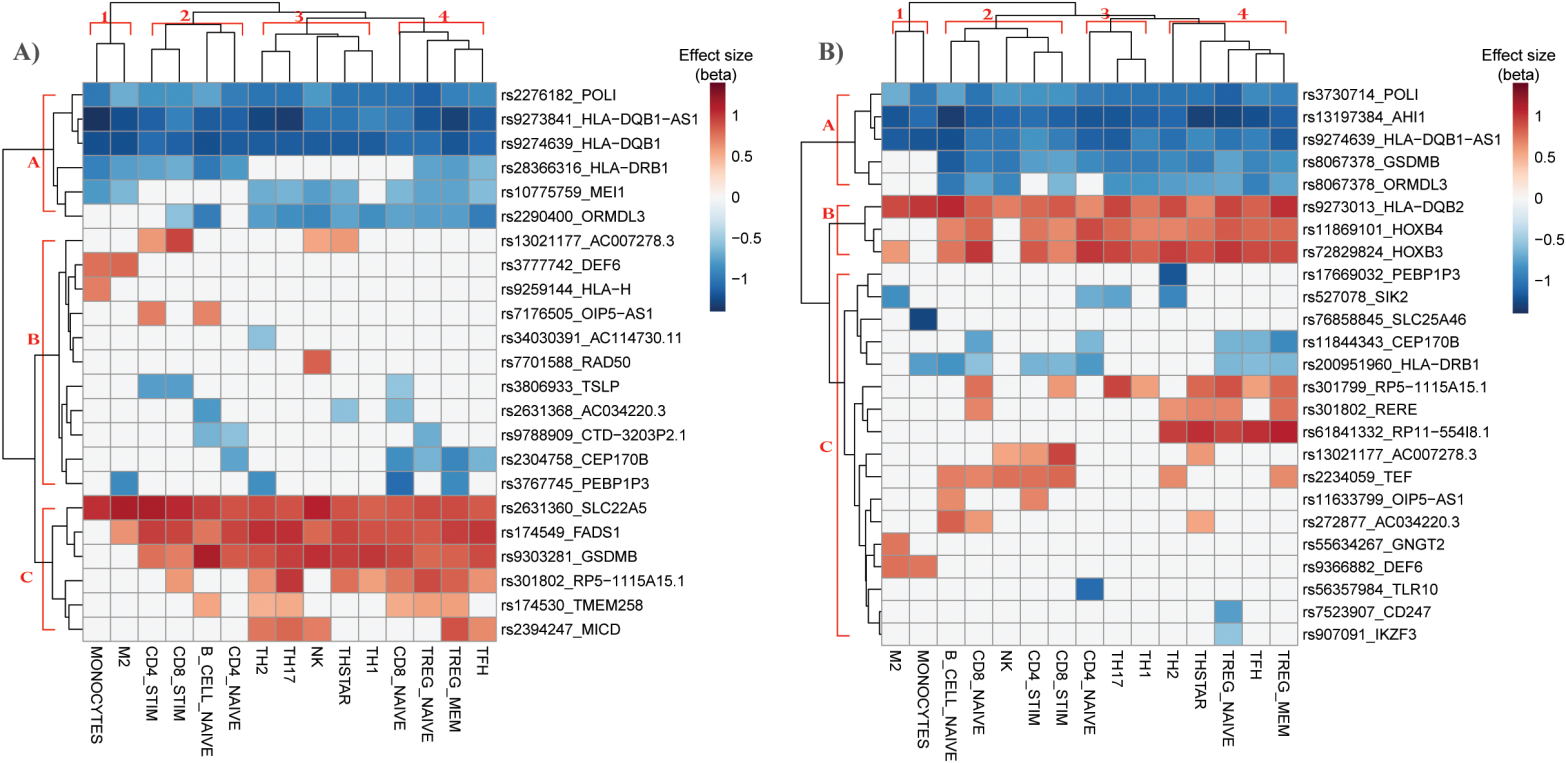
Hierarchical clustering of eQTL-gene pairs causally associated with asthma in immune cell types identifies potential driver cell types for asthma risk. Hierarchical clustering of DICE immune cell types (columns) and causal asthma eQTL-genes (rows) identified in **(A)** lung and **(B)** blood. Color represents the effect size of the eQTL’s alternate allele obtained from DICE.

Various immune cell types have been associated with the development of asthma. Therefore, we mapped the eQTL-gene pairs causally associated with unspecified asthma from lung and blood onto immune cell type-specific cis-eQTL data to prioritize potential driver immune cell types. Cell type-specific cis-eQTL data (not spatially constrained) was obtained from the Database of Immune Cell eQTLs (DICE) across 15 immune cell types (see methods). Of the 63 eQTL-gene pairs causally associated with unspecified asthma in the lung, 23 were identified as cis eQTL-gene pairs across 15 immune cell types (**Figure 3A**). To distinguish the direction of effect in different cell populations, we performed unsupervised hierarchical clustering of effect sizes (beta) of cis-eQTLs across the cell types. In the lung, hierarchical clustering identified three main clusters of causal asthma eQTL-gene pairs: causal eQTL-gene pairs with A) negative, B) both positive and negative, and C) positive effect sizes (**Figure 3A**). Additionally, four cell-type clusters (clusters 1-4) were enriched for various causal eQTL-gene pairs. For example, cluster 1 is the monocyte cluster and is enriched for causal eQTL-gene pairs from cluster A. Cluster 2 is primarily CD8+ and CD4+ T cells, and cell clusters 3 and 4 are predominantly CD4+ T memory subtypes.

Of the 65 single-IV causal eQTL-gene pairs identified in blood, 25 were also identified across the 15 DICE immune cell types (**Figure 3B**). Hierarchical clustering identified three main clusters of causal asthma eQTL-gene pairs (A-C) and four cell-type clusters (clusters 1-4).

### 3.4 Different genes are causally associated with the development of COA in lung and blood

**Figure 4:**
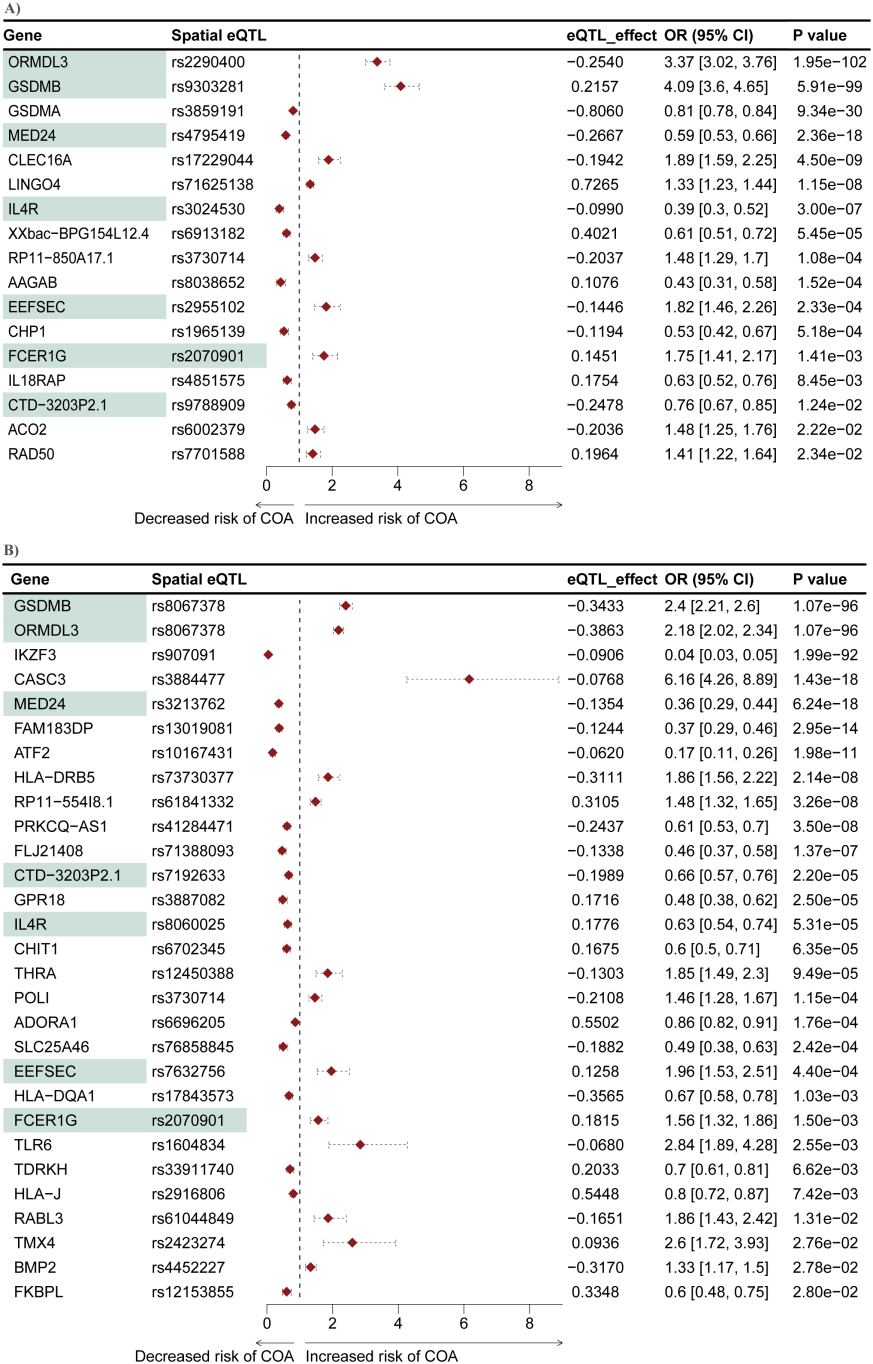
Mendelian randomization identifies seven genes that are causally associated with COA in both the L-GRN and B-GRN. 17 and 29 COA causal genes were identified in the **(A)** L-GRN and **(B)** B-GRN, respectively. Genes (n = 7) and spatial eQTL-gene pairs (n = 1) shared across both GRNs are shaded in green. Horizontal dotted lines illustrate the 95% CI for each odds ratio.

A two-sample MR analysis was conducted to test whether genes within the L-GRN and B-GRN had a causal association with the development of COA. The altered expression of 17 and 29 genes was causally associated with COA using the Wald ratio method in the L-GRN and B-GRN, respectively (**Figure 4**). No statistically significant genes with multiple IVs were identified in either tissue using the IVW method. Of the identified genes in blood, *ATF2*, *BMP2*, and *RABL3* were regulated by trans-intrachromosomal spatial eQTLs (eQTLs whose target genes are >1Mb away within the same chromosome), the remaining 34 genes were regulated in cis. Seven genes (*ORMDL3*, *GSDMB*, *MED24*, *IL4R*, *EEFSEC*, *FCER1G*, and *CTD-3203P2.1*) were shared across the L-GRN and B-GRN and had the same direction of effect. However, the shared genes had larger odds ratios in the lung when compared to blood, except for *IL4R*, which had larger odds ratios in blood. Apart from rs2070901-*FCER1G*, the genes identified as being causal in both tissue GRNs were associated with different spatial eQTLs in each tissue. Pathway enrichment analysis of the 39 genes causally associated with COA identified enriched pathways including, T-cell activation and response to other organisms (**Supplementary Figure S4**).

### 3.5 Mendelian randomization putatively implicates ten genes with the development of AOA

**Figure 5:**
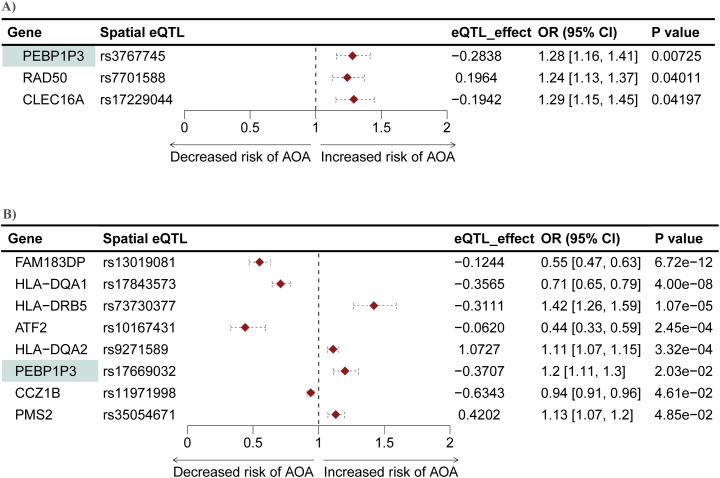
Mendelian randomization identifies genes causally associated with AOA in both the L-GRN and B-GRN. Three and eight genes causally associated with AOA were identified in the **(A)** L-GRN and **(B)** B-GRN, respectively. PEBP1P3 (shaded green) was shared across both GRNs. 95% CI of each odds ratio is shown as horizontal dotted lines.

We conducted another two-sample MR analysis to investigate potential causal associations between genes within the L-GRN and B-GRN and the development of AOA. We identified three and eight genes that were causally associated with the development of AOA in the L-GRN and B-GRN, respectively (**Figure 5**). Of the identified putatively causal genes, only *ATF2* was regulated by trans-intrachromosomal spatial eQTLs, the remaining genes were regulated in cis. *PEBP1P3*, was identified across both tissues using the Wald ratio method but was associated with different spatial eQTLs in each tissue (*i.e*., rs3767745 in the lung and rs17669032 in the blood). The direction of effect of *PEBP1P3* was maintained across tissues. Similar to COA's MR analysis results, no statistically significant causal genes with multiple IVs were identified using the IVW method. Pathway enrichment analysis on the ten genes causally associated with the development of AOA identified enriched pathways including lymphocyte activation and MHC protein complex (**Supplementary Figure S4**).

### 3.6 Mendelian randomization identifies 21 genes as being causally associated with unspecified asthma and COA, but not AOA

To better understand the extent to which the underlying genetic risk of COA and AOA overlap, we compared the causal genes and their enriched pathways for each phenotype. Six causal genes were shared between COA and AOA (*i.e*., *RAD50*, *CLEC16A*, *ATF2*, *HLA-DRB5*, *FAM183DP*, and *HLA-DQA1* [**Supplementary Figure S3**]). Of the identified shared genes, two are lung-specific (*i.e*., *RAD50* and *CLEC16A* [**Figure 3B**]), and four are blood-specific (*i.e*., *ATF2*, *HLA-DRB5*, *FAM183DP*, and *HLA-DQA1*).

**Figure 6:**
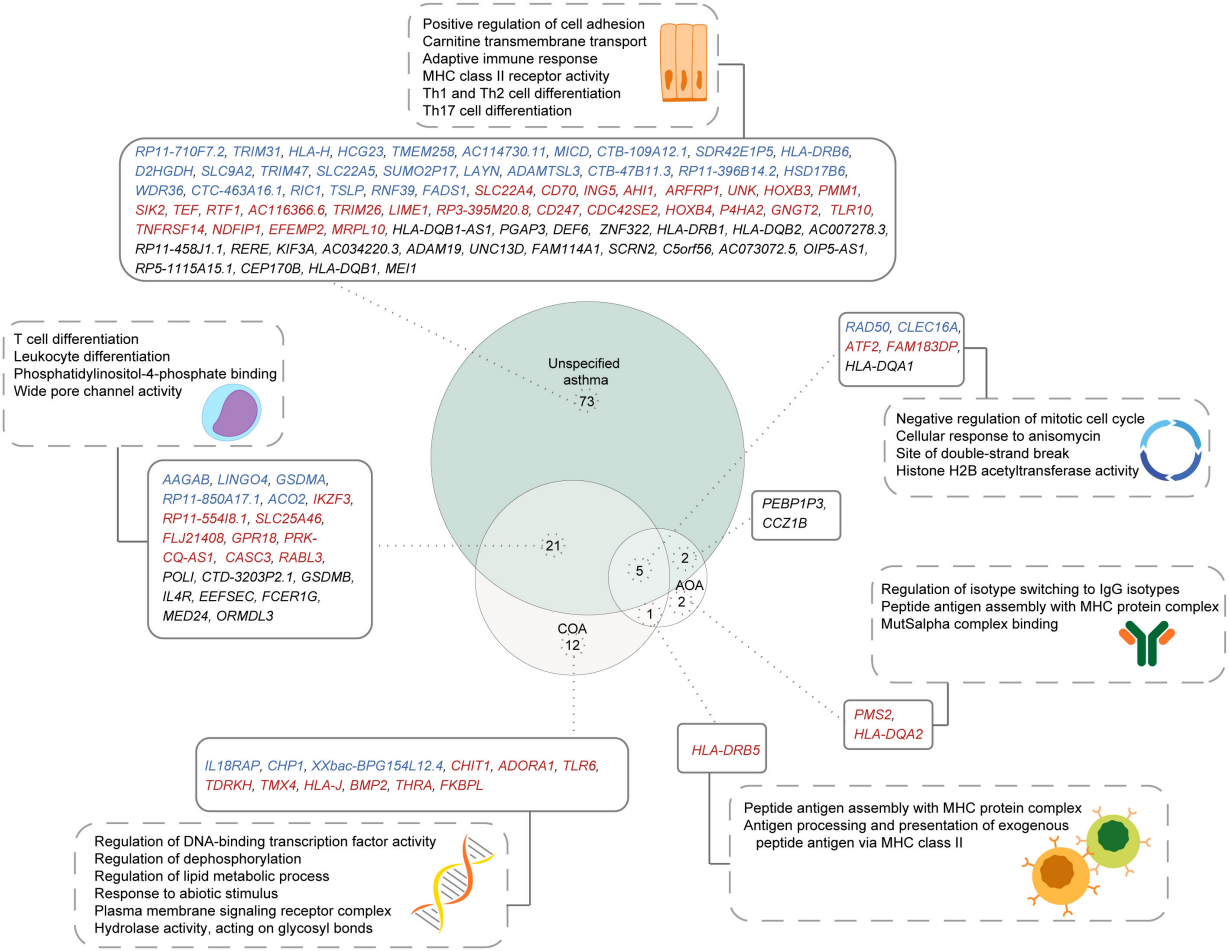
Shared and unique putative asthma causal genes exist for different asthma subtypes. A comparison of genes causally associated with each of unspecified asthma, COA, and AOA, along with their enriched pathways. Genes specific to the L-GRN and B-GRN are shown in blue and red, respectively. Genes shared across GRNs are in black. Statistically enriched (p < 0.05) pathways associated with each gene set are shown in dashed rectangles.

Most population studies on asthma have involved a diverse population of asthmatics defined using a broad asthma definition (38, 39). To understand the implications of this diversity on the identification of putative causal genes, we compared the causal genes and pathways identified for unspecified asthma with those identified for COA and AOA. Among the 101 genes identified as causally associated with unspecified asthma, 28 were identified as being causally associated with COA and/or AOA (**Figure 6**). Of the 28 shared genes, five genes were shared between all subtypes, 21 genes were exclusively shared between COA and unspecified asthma, and two genes were exclusively shared between unspecified asthma and AOA. Additionally, 73, 12, and two genes were unique to each of unspecified asthma, COA, and AOA, respectively. *HLA-DRB5* was shared exclusively between COA and AOA. Gene set enrichment analysis identified the five genes shared between all asthma subtypes as being enriched in pathways including negative regulation of the mitotic cell cycle (**Figure 6**). By contrast, the 22 genes exclusively shared between unspecified asthma and COA were enriched for pathways including T cell differentiation (**Figure 6**).

## 4 Discussion

We identified genes whose altered expression in lung or blood is putatively causally associated with unspecified asthma and two age-specific asthma presentations. Specifically, two-sample MR analysis identified 101, 39, and ten genes causally linked to the development of unspecified asthma, COA, and AOA, respectively, with 29 genes being shared across some or all subtypes. Of the 101 genes identified as causally associated with unspecified asthma, 32 were shared across the lung and blood. These shared genes were associated with different magnitudes of effects on asthma. Nevertheless, 27 of the shared genes maintained the direction of effect across tissues. The consistent direction of effect suggests that these 27 genes may play a role in asthma that spans multiple physiological processes. Accordingly, targeting these genes might result in a greater therapeutic outcome for asthma. The identification of five genes that are causally associated with unspecified asthma as having opposite directions of effects in either tissue emphasizes the importance of interpreting results in their tissue-specific context (40).

Valette et al. identified 50 causal asthma genes in blood using non-spatial eQTLs as IVs (8). Our study replicated 19 of these genes to be causally associated with unspecified asthma. Of the shared genes, we identified six in both the lung and blood (*i.e.*, *GSDMB*, *RERE*, *PGAP3*, *UNC13D*, *MEI1*, and *SCRN2*), three were exclusive to the lung (*i.e.*, *TMEM258*, *SLC22A5*, and *FADS1*), and ten were identified only in blood (*i.e.*, *IKZF3*, *SLC22A4*, *ING5*, *AHI1*, *SIK2*, *TEF*, *HOXB4*, *GNGT2*, *TLR10*, and *EFEMP2*). Notably, two of the identified genes are targets of known asthma drugs (*i.e.*, *TSLP* [Tezepelumab] and *IL4R* [Dupilumab] (41, 42)). For COA, 18 genes identified in our study were previously prioritized as candidate risk genes for COA (*ORMDL3*, *GSDMB*, *MED24*, *CLEC16A*, *LINGO4*, *IL4R*, *IL18RAP*, *RAD50*, *MED24*, *HLA-DRB5*, *ADORA1*, *SLC25A46*, *HLA-DQA1*, *TLR6*, *POLI*, *FKBPL*, *RAD50*, and *TDRKH*) by Pividori et al. (17) and/or Ma et al. (43). Of these, three genes (*RAD50, CLEC16A*, and *HLA-DQA1*) were also identified as candidate risk genes for AOA in both our analysis and that of Pividori et al. (18).

17q12-21 is a genomic region consistently replicated as the most statistically significant locus in asthma GWASs (44). Among the protein-coding genes causally associated with unspecified asthma (n=73), seven were located within 17q12-21. Six of those putatively causal genes located in 17q-12-21 (*i.e., IKZF3, GSDMB, ORMDL3, GSDMA, MED24*, and *CASC3*) were shared with COA, and *THRA* was unique to COA. Notably, we did not identify any genes within the 17q12-21 locus as being causally associated with the development of AOA. This is consistent with onset-specific asthma association studies indicating that disease-associated SNPs within 17q12-21 are specific to COA (17, 44). Moreover, we identified six genes as being causally associated with the development of both COA and AOA. Interestingly, these shared genes displayed larger odds ratios for COA than for AOA. This observation, coupled with the greater number of genes identified as causally associated with COA compared to AOA, corresponds with current estimates that the SNP-based heritability of COA is approximately threefold that of AOA (17, 45).

In our comparison of causal genes identified for unspecified asthma, COA, and AOA, we observed both shared and subtype-specific putatively causal genes. It is important to emphasize that the GWAS used for the identification of genes causally associated with unspecified asthma involved a heterogeneous population of asthmatics defined using a broad asthma definition (8). The identification of genes or pathways specific to COA and AOA and not unspecified asthma could be due to the lack of subtype representation and insufficient statistical power to detect subtype-specific variants in the unspecified asthma sample. This possibly emphasizes the importance of considering the different asthma phenotypes in population studies.

Pathway enrichment analysis of the genes causally associated with unspecified asthma, COA, or AOA revealed both shared and unique yet interrelated pathways. For example, pathways involved in T cell function (*e.g*., T cell activation, MHC class II protein complex assembly, allograft rejection, and graft-versus-host disease) were associated with all subtypes. However, T cell differentiation was enriched only in unspecified asthma and COA. This observation may be attributed to the initial priming of the immune system during early life (46). Consistent with this, a developmental switch in T cell differentiation has been reported to occur in childhood (47). Moreover, studies indicate that the composition of the naïve T cell subset, which differentiates to T memory cells, undergoes dynamic change from childhood to adulthood in health (48). This transition involves a gradual reduction in the naïve T cell subset accompanied by a gradual expansion of the T memory subset (48). Furthermore, a higher ratio of T regulatory cells (which suppress T cell activation) to T memory cells has been identified in infancy compared to adulthood, suggesting tighter control of T cell activation and differentiation in early life (49, 50). Dysregulation of this control might be a contributing factor to the development of COA (51).

In line with this, our cell type-specific analysis on the 15 immune cell types shows that most causal eQTL-gene pairs map to the T-regulatory subset. However, this analysis was restricted to unspecified asthma due to data sparsity at the COA and AOA levels. Future analyses could thus include additional in-depth investigations that integrate asthma GWAS data with scRNA-seq data to identify subtype-specific driver cell types and critical risk genes (52). Tools such as the scPagwas method could be used for such an analysis (53), and a similar approach has been shown to identify critical immune cell types relevant to severe COVID-19 (54). These analyses will really come into their own when case/control scRNAseq data becomes available at the asthma subtype level.

Among the pathways enriched within the causal gene sets, negative regulation of the mitotic cell cycle was enriched in all three asthma subtypes. Given that the cell cycle (and DNA synthesis) facilitates the remodeling of genes from an inactive to an active state (55, 56), this finding could pertain to a role in controlling immune cell differentiation. Notably, T cell differentiation was identified as enriched exclusively for COA and unspecified asthma, while unspecified asthma was specifically enriched for “Th1 and Th2 cell differentiation” and “Th17 cell differentiation”. Conversely, the enrichment of Th1 and Th17-associated pathways in unspecified asthma but not COA and AOA aligns with their established associations with non-type 2 inflammation (one of the two inflammatory profiles of asthma), which encompasses smoking-associated, obesity-associated, and very late-onset asthma (57). Although not directly investigated in our analysis, it is plausible that these asthma subtypes were represented within the unspecified asthma cohort to some extent. Conversely, COA and AOA, particularly the allergic phenotype, fall under the type 2/T2 inflammatory profile, involving cells including Th2 and innate lymphoid cells (58). These findings highlight the need to take into account the different molecular pathways and inflammatory profiles associated with different asthma subtypes when considering therapeutic options.

Airway remodeling is a characteristic feature of asthma. The airway epithelium barrier, in particular, is compromised in asthmatic patients with changes involving detachment of ciliated cells, increased permeability to environmental allergens, increased goblet cell numbers and downregulated expression of junctional proteins, which are important for cell-cell adhesion (59–62). We have identified pathways relevant to epithelial barrier function (*i.e.*, positive regulation of cell adhesion) as being enriched in the genes causally associated with unspecified asthma. Additionally, the pathway “wide pore channel activity” was exclusively enriched between unspecified asthma and COA. This enrichment is driven by *GSDMB* and *GSDMA*, members of the GSDM gene family that play an important role in antibacterial immune defenses through the formation of pores in bacterial membranes (63). This is consistent with the high levels of expression of GSDM genes, particularly *GSDMB*, in ciliated airway epithelial cells and underscores its antibacterial functions at organism barriers (64). These findings emphasize the critical role of events at the airway epithelial surface in the development of asthma, potentially indicating a more prominent role in COA compared to AOA.

Among the pathways specifically enriched for AOA, we identified regulation of isotype switching to IgG isotypes. Isotype switching refers to the process by which B cells change the class of antibodies they produce without losing antigen specificity. This finding is consistent with studies showing that among primary immunodeficiencies, immunoglobulin G3 deficiency was the most associated with asthma exacerbation in adults, where regular intravenous immunoglobulin (IVIg) therapy successfully prevented exacerbations of asthma (51, 65).

This study is not without limitations. Firstly, the causal associations identified through MR are putative and further experimental evidence is required to validate them. Secondly, we did not consider other asthma subtypes in our analysis (*e.g.*, severe, atopic, non-atopic asthma, etc.); this should be taken into consideration when interpreting the enriched pathways identified by our analysis. Thirdly, the GRNs used in this analysis portray a static view of interactions within specified cell lines at specific time-points, which are susceptible to change. Fourthly, the Hi-C datasets used to generate the GRNs do not originate from the same GWAS participants and are not age-matched. Accordingly, if onset-specific chromatin interactions exist, these could be missed by our analysis. Lastly, datasets used in this analysis are derived primarily from individuals of European ancestry (*e.g.*, GTEx and the UK Biobank). Therefore, the results may not be generalizable to diverse populations. Notwithstanding these limitations, we identify putative genes causally associated with unspecified asthma, COA, and AOA, and highlight pathways potentially involved in age-specific developments of asthma. The practical implication of this study is that targeting treatments based on a patient’s asthma subtype and genetic profile may enhance therapeutic efficacy and reduce the risk of adverse drug responses. Notably, leveraging genetic evidence in the identification of gene targets is estimated to double the success rate of clinical development in drug discovery (66, 67). Consequently, we propose that the causal genes identified in this analysis hold promise as potential drug targets, emphasizing the need to consider the asthma subtype in the development of asthma drugs.

## Data Availability

The datasets presented in this study can be found in online repositories. The names of the repository/repositories and accession number(s) can be found below: Figshare repository [https://doi.org/10.6084/m9.figshare.20205644.v1 and https://doi.org/10.17608/k6.auckland.17067953.v1].
