# Supplementary material for: Unspecified asthma, childhood-onset, and adult-onset asthma have different causal genes: a Mendelian randomization analysis
Source: Front Immunol. 2024 Nov 19;15:1412032. doi: 10.3389/fimmu.2024.1412032 (PMC11611866; doi:10.3389/fimmu.2024.1412032)
Supplement: Supplementary file 1 [file DataSheet1.docx]

Supplementary Material

_­_**1 Supplementary Data**

Supplementary Data 1 – 2 are available at frontiers online.

The lung spatial gene regulatory network: [10.6084/m9.figshare.20205644.v1](https://doi.org/10.6084/m9.figshare.20205644.v1)

The whole blood spatial gene regulatory network: [10.17608/k6.auckland.17067953.v1](https://doi.org/10.17608/k6.auckland.17067953.v1)

_­_**2 Supplementary Figures**


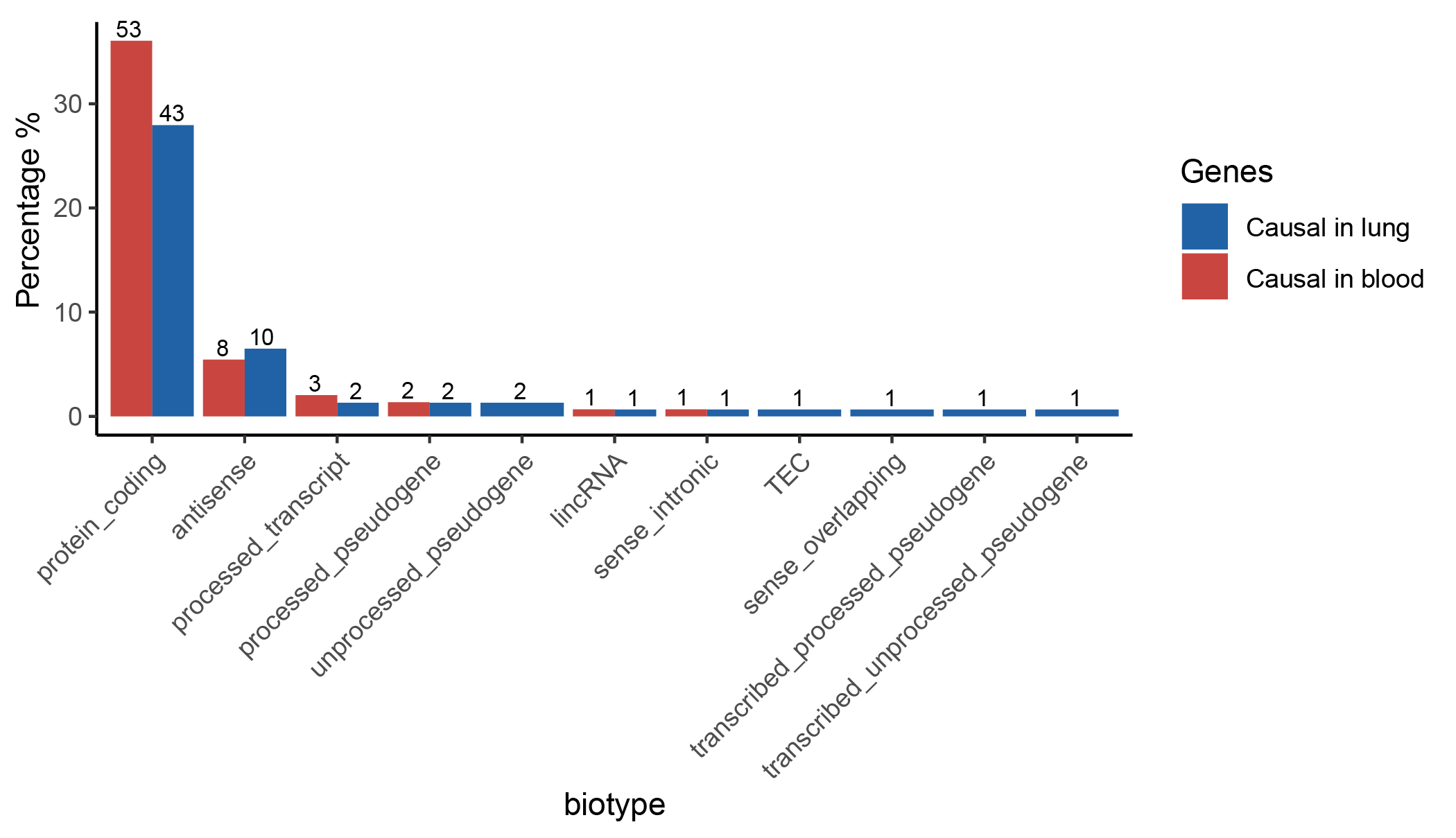


**­Supplementary Figure 1**. Biotype annotation of the 65 asthma causal genes in lung (blue) and the 68 asthma causal genes in blood (red).


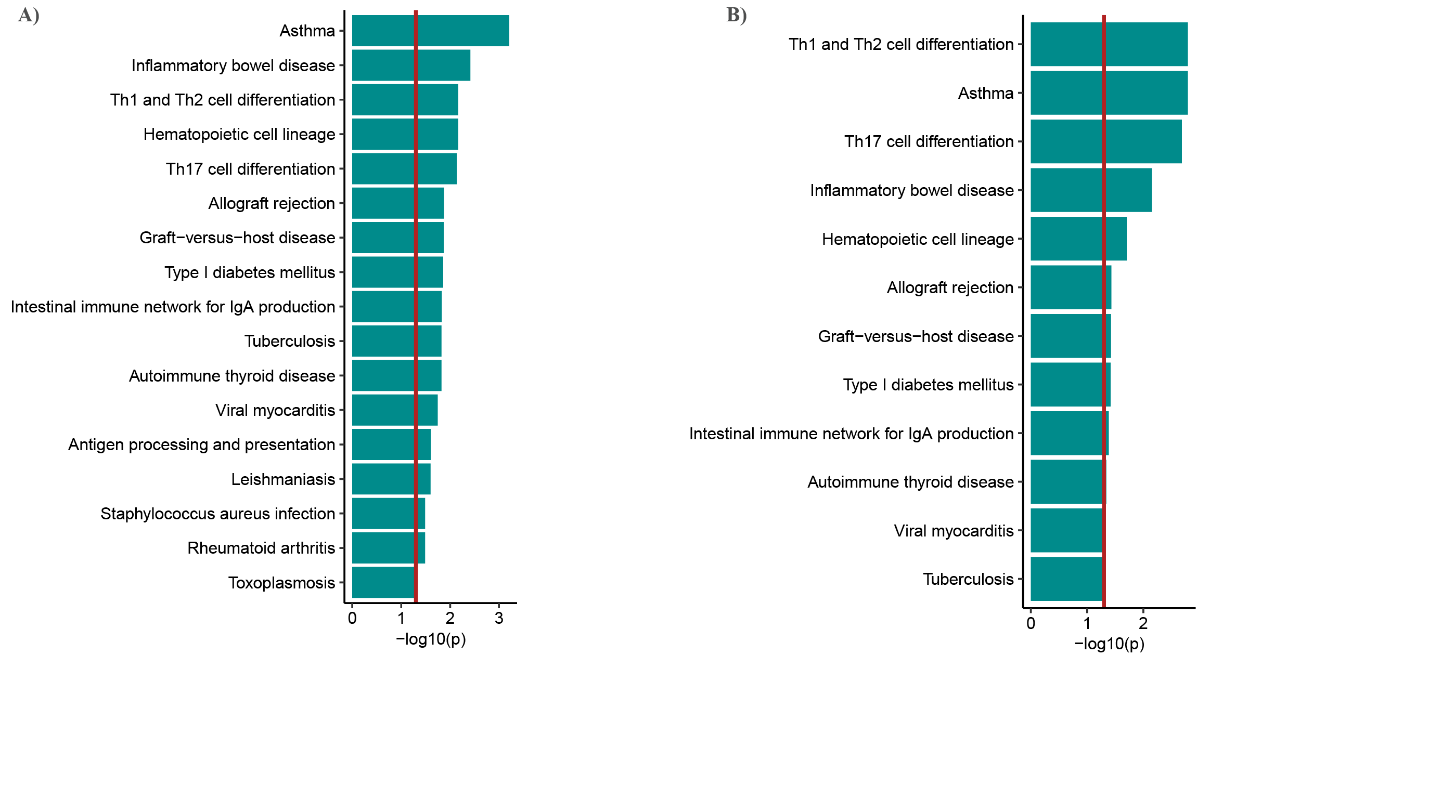


**­Supplementary Figure 2. Asthma causal genes are enriched for KEGG pathways including asthma and T-cell associated traits.** GO enrichment analysis of **A)** L-GRN and **B)** B-GRN asthma causal genes in KEGG pathways. The red line indicates a p-value of 0.05.


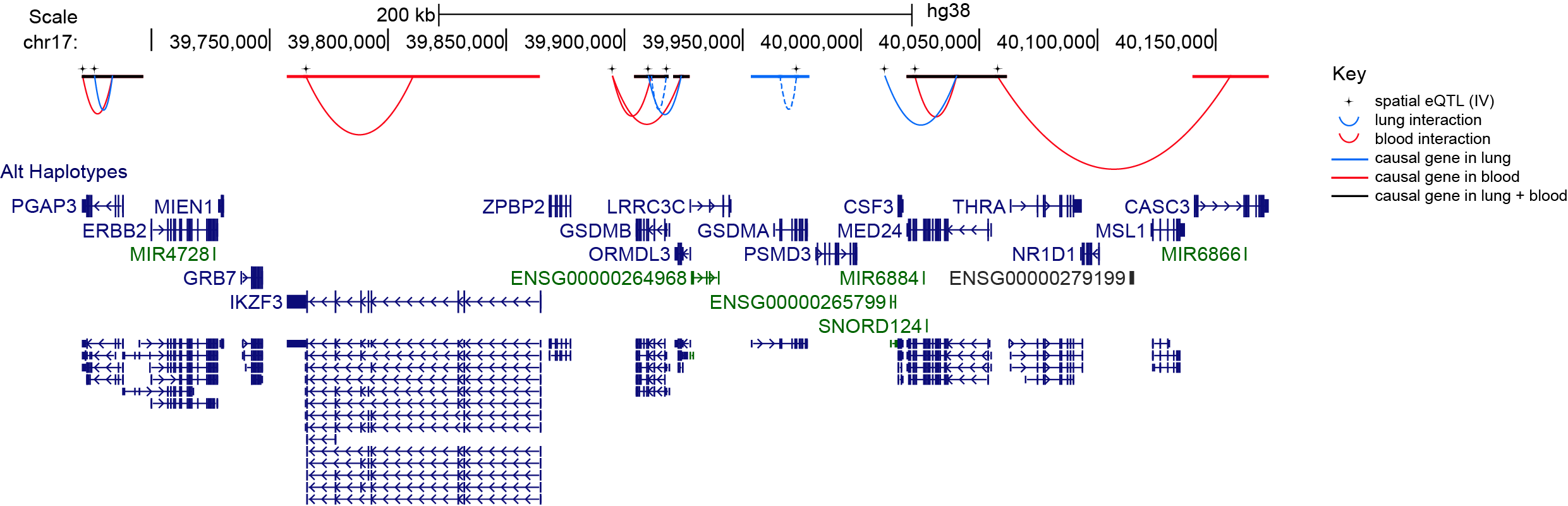


**­Supplementary Figure 3. Seven asthma causal protein-coding genes located within 17q12-21 have distinct regulatory patterns.** UCSC browser genome tracks spanning chr17:39,670,900-40,172,190 show 11 spatial eQTLs (IVs) regulating seven protein-coding genes that are causal for asthma in either lung (blue) blood (red) or both tissues (black).


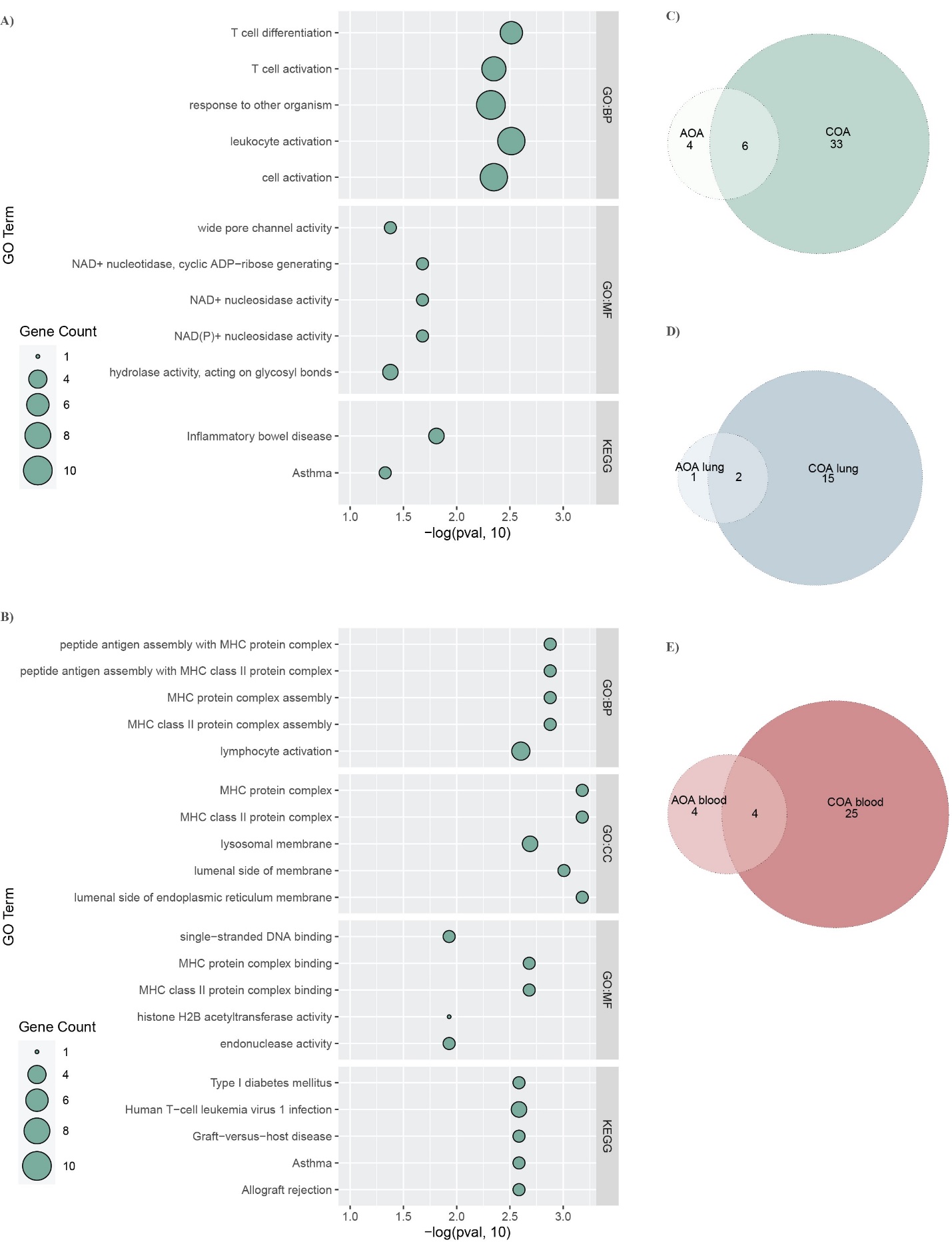


**­Supplementary Figure 4. Six genes are causally associated with both COA and AOA by Mendelian randomization.** Pathway enrichment analysis of genes causally associated with **A)** COA and **B)** AOA. BP = biological processes, MF = molecular functions, CC = cellular components. Shared and unique genes identified as causally associated with **C)** COA and AOA identified in the **D)** L-GRN and **E)** B-GRN.
